# Long-term clinical outcomes of allograft-prosthetic reconstruction for tumours of the extremities

**DOI:** 10.1007/s00264-026-06819-x

**Published:** 2026-04-23

**Authors:** Philip T.J. Sanders, Sjors F. van de Vusse, Giovanni Simonis, Marta Fiocco, Michiel A.J. van de Sande, P.D. Sander Dijkstra, Michaël P.A. Bus

**Affiliations:** 1 Department of Orthopaedic Surgery, Leiden University Medical Center, Leiden, Netherlands; 2 Department of Biomedical Data Sciences, Leiden University Medical Center, Leiden, Netherlands; 3 Mathematical Institute, Leiden University, Leiden, Netherlands

**Keywords:** Allograft-prosthetic composite, Bone tumour reconstruction, Orthopaedic oncology, Limb salvage

## Abstract

**Purpose:**

Allograft-prosthetic composites (APC) are used to reconstruct large periarticular defects following tumour resection, with potential advantages especially restoration of bone stock and ligamentous reattachment. While short- and mid-term outcomes have been reported on extensively, long-term clinical results remain limited. This study evaluated the incidence of mechanical and non-mechanical complications, risk factors for complications, and the cumulative incidence of reconstruction failure following APC reconstruction for extremity tumours with a minimum follow-up of ten years.

**Methods:**

We retrospectively reviewed 64 APC with at least ten years follow-up in our centre. Predominant diagnoses were osteosarcoma (40%) and chondrosarcoma (28%). Reconstructions involved the proximal femur (39%), distal femur (22%), proximal tibia (23%) and proximal humerus (16%). Median follow-up was 24.5 years (95%CI 23.6–25.4).

**Results:**

Instability occurred in nine reconstructions (14%). Non-union was observed in nine reconstructions (14%). Implant loosening occurred in seven reconstructions (11%) after a median of 14 years (range 2–18 years). Allograft collapse occurred in 13 reconstructions (20%) after a median of three years (range 1–15). Infection developed in five reconstructions (8%). Cumulative incidence of mechanical failure at five, ten and 25 years was 15.6% (95%CI 6.6–24.6), 21.9% (95%CI 11.6–32.1) and 28.6% (95%CI 17.2–39.9), respectively.

**Conclusions:**

APC are associated with a considerable risk of both early and late complications. Non-union and infection predominate in the early postoperative period, whereas aseptic loosening and fractures are the main causes of late failure, occurring up to 18 years after surgery. These findings suggest that the routine use of APC for periarticular reconstruction after tumour resection should be reconsidered.

## Introduction

Primary malignant bone tumours most frequently affect the epimetaphyseal region of the long bones [1, 2]. Because these tumours are primarily treated with *en bloc* resection, joint reconstruction is commonly indicated [3]. Several techniques have been described for periarticular reconstructions, including osteoarticular allografts, endoprostheses, and a combination of the aforementioned; allograft-prosthetic composites (APC).

Osteoarticular allografts offer the possibility to reattach tendons and to restore bone stock [4, 5]. However, previous authors have raised concern about the longevity of osteoarticular allografts, as they have been associated with a considerable risk of mechanical failure and degenerative joint disease [6–8]. To address the risk of these degenerative changes, APC were introduced as an alternative method of reconstruction in the early 1980’s [9]. Studies however showed that APC were not free from allograft-related complications, including loosening, fractures and allograft-host nonunion [10, 11]. During recent decades, endoprostheses gradually replaced osteoarticular allografts and APC as the technique of choice, because endoprosthetic systems rapidly refined and offer the advantages of quick recovery and early weight-bearing [12–14].

Previous studies on APC reported acceptable reconstruction survival rates (68–100%) [15, 16] and satisfactory functional results (Musculoskeletal Tumour Society functional outcome scores of 82–88%) at mid-term (range 56–76 months)[16, 17]. However, study populations were generally small. To our knowledge, there are no studies that report long-term outcomes of APC. Therefore, we have only limited understanding of the durability and failure mechanisms of APC on the long term. With this study, we aimed to evaluate: (1) the incidence of mechanical and non-mechanical complications; (2) risk factors for complications, and (3) the cumulative incidence of mechanical failure at five, ten and 25 years for APC for primary bone tumours in the extremities, with a minimum follow-up of ten years.

## Materials and methods

We queried our institutional databases to identify all patients aged ≥ 16 years who underwent allograft-prosthetic composite reconstruction following resection of a primary bone tumour in the extremities (femur, proximal tibia and proximal humerus) between 1989 and 2016. Patients were followed for a minimum of ten years or until death. In the initial period under study, we regularly performed APC after *en bloc* resection of a primary bone tumour around the hip, knee or shoulder. Alternative treatments at that time included osteoarticular allografts and endoprosthetic reconstruction. If the adjacent joint could be preserved, an intercalary resection was preferred.

We identified 57 patients (32 male, 56%) with a total of 64 APC (Table 1,2). Of the 64 reconstructions, 16 (25%) were performed as a revision of a failed previous reconstruction (Table 3). Median age at surgery was 34 years (range 16–68). Twenty-five tumours (39%) were located in the proximal femur, 14 (22%) in the distal femur, 15 (23%) in the proximal tibia and ten (16%) in the proximal humerus. Predominant diagnoses were osteosarcoma (n = 23, 40%) and chondrosarcoma (n = 16, 28%). Thirty-one patients (54%) received (neo)adjuvant chemotherapy around the period of APC implantation, five (9%) had adjuvant radiation therapy.

**Table 1:** Patient details

	**N (%)**
*Patients*	*57 (100)*
Sex	
Male	32 (56)
Female	25 (44)
American Society of Anaesthesiologists (ASA) score [32]	
ASA 1	25 (44)
ASA 2	31 (54)
ASA 3	1 (2)
Diagnosis	
Osteosarcoma	23 (40)
Chondrosarcoma	16 (28)
Ewing sarcoma	4 (7)
Pleomorphic undifferentiated sarcoma	10 (18)
Others	4 (7)
Adjuvant therapy	
Neo-adjuvant chemotherapy	31 (54)
Adjuvant chemotherapy	30 (53)
Neo-adjuvant radiotherapy	1 (2)
Adjuvant radiotherapy	4 (7)
Pathological fracture at presentation	9 (16)

**Table 2:** Reconstruction details

	Proximal femur N (%)	Distal femur N (%)	Proximal tibia N (%)	Proximal humerus N (%)
Osteosynthesis type				
Short plate	12 (48)	1 (7)	6 (40)	5 (50)
Long plate	1 (4)	7 (50)	5 (33)	1 (10)
Multiple plates	6 (24)	5 (36)	4 (27)	1 (10)
Cerclage	1 (4)	0 (0)	0 (0)	1 (10)
None	5 (20)	1 (7)	0 (0)	2 (20)
Type of fixation				
Plate only	8 (32)	13 (93)	15 (100)	3 (30)
Plate and implant bridging	6 (24)	0 (0)	0 (0)	4 (40)
Implant bridging	11 (44)	1 (7)	0 (0)	3 (30)
Allograft cementation	11 (44)	14 (100)	15 (100)	6 (60)
Intramedullary strut grafting	7 (28)	11 (79)	8 (53)	2 (20)

**Table 3:** Procedures performed before APC

Procedure	Reconstruction	Number	Reasons for reconstruction failure
*En bloc* resection	Allograft-prosthetic composite	7	Allograft fracture(n = 3), non-union(n = 1), loosening(n = 1), hardware fracture(n = 1), infection(n = 1)
	Endoprosthesis (KMFTR, Stryker Howmedica Osteonics, Kiel, Germany)	1	Hardware failure
	Intercalary allograft	1	Non-union
	Osteoarticular allograft	3	Degenerative joint disease (n = 2), allograft fracture (n = 1)
Curettage	Cancellous bone grafting	3	Tumour recurrence
Osteosynthesis	Dynamic hip screw	1	‘Whoops’ procedure (performed elsewhere)

Median follow-up was calculated using the reverse Kaplan–Meier method, and was equal to 24.5 years (95% confidence interval [CI], 23.6–25.4). At final follow-up, 26 patients (46%) were alive, 22 patients (39%) had died of disease, and nine patients (16%) had died of other causes. At ten and 15 years follow-up, 37 (65%) and 29 (51%) were alive, respectively. None of the patients were lost to follow-up.

Allografts were harvested during post-mortem tissue donation and stored at −80 °C by our national bone bank. The grafts were either not subjected to additional sterilization, or sterilized with low-dose gamma radiation (< 25 kGy). Prior to surgery, all patients received prophylactic cephalosporin antibiotics, and these were continued for one to five days. During tumour resection, the allograft was thawed in saline with antibiotics.

**Fig. 1:**
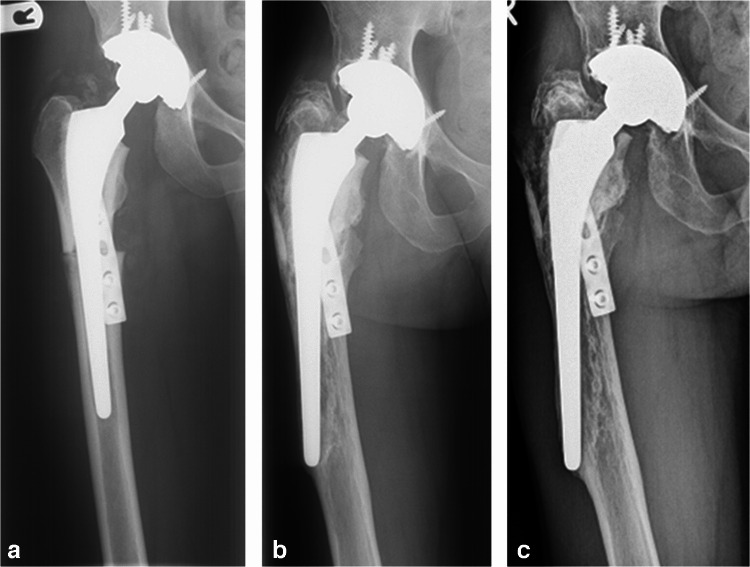
APC reconstruction of the proximal femur following resection of an Ewing sarcoma in an 18-year-old male patient. **A**: anteroposterior view one month after the primary procedure, showing a femoral stem that bridges the osteotomy line, and a short five-hole compression plate. **B**: at five months, there are signs of allograft resorption and there is evident migration of the femoral component. **C**: seven years after the primary procedure, showing collapse of the allograft and implant migration. Conversion to an endoprosthesis was performed

**Fig. 2:**
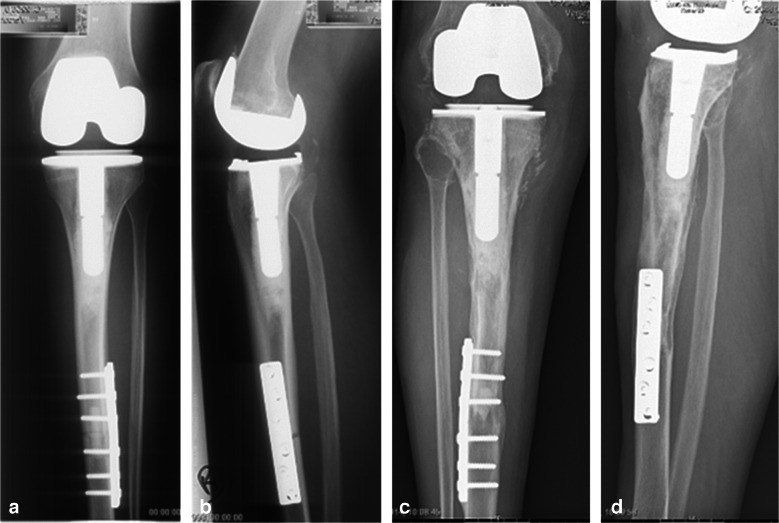
APC reconstruction of the proximal tibia following resection of a grade 1 chondrosarcoma. **A** and **B**: direct postoperative anteroposterior and lateral radiograph, showing reconstruction with a conventional knee replacement system, fixation of the allograft with a plate and additional intramedullary strut grafting. **C** and **D**: anteroposterior and lateral radiographs taken 18 years after the index procedure. The patient did not experience any complications. There is sound incorporation of the allograft and there are no signs of implant loosening

Median reconstruction length was 19 cm (8–43) for the proximal femur, 17 cm (13–27) for the distal femur, 12 cm (8–20) for the proximal tibia and 12 cm (8–22) for the proximal humerus. All osteotomies were transverse. In 56 reconstructions (88%), plate osteosynthesis was performed to stabilize the allograft, most often using two short 4-hole dynamic compression plates (Table 2, Fig. 1). In most cases, conventional arthroplasty systems were used; Mallory-Head (Biomet, Warsaw, Indiana, US) for the hip, Insall-Burstein (Zimmer, Warsaw, Indiana, US) for the knee and the Bio-Modular humeral prosthesis (Biomet, Warsaw Indiana, US) for the shoulder. The prosthesis was cemented into the allograft in 11 of 25 (44%) proximal femoral reconstructions, six of ten (60%) proximal humeral reconstructions and in all knee replacements. In 25 reconstructions (39%), a stemmed implant bridged the osteotomy to stabilize the construct (Fig. 1). Intramedullary strut grafting was performed in 28 (44%) reconstructions (Fig. 2), with the aim to reinforce the construct and to promote bone healing (Table 2).

Resection margins were clear in 55 patients (96%) and inconclusive in one (2%). One patient (2%) had intentional intralesional surgery (giant cell tumour of bone). Median postoperative hospital stay was 15 days (range 7–45). Weight-bearing was allowed when the reconstruction was deemed stable on the basis of physical examination and conventional radiographs. Follow-up routinely included functional and radiographic examination with conventional radiographs. If a recurrence was suspected, additional magnetic resonance imaging was performed. Complications and failures were classified according to Henderson et al. [18]. Non-union was defined as surgical intervention to facilitate union of an osseous junction, at least six months after primary surgery [19]. Aseptic loosening was defined as migration of the prosthesis on conventional radiographs or CT imaging and/or halo formation on CT imaging, in the absence of infection. Failure was defined as complete removal of the construct.

### Statistical analysis

A competing risks model with three competing events was used to estimate the cumulative incidence of mechanical failure and failure due to infection with patient mortality as a competing event [20, 21]. Risk factors associated with loosening, collapse, and infection were assessed by using cause specific Cox proportional hazard regression models. Statistical analysis concerning the competing risk model was performed in the R environment with the mstate library [20]; remaining analyses were performed with SPSS 29.0 (IBM Corp, Armonk, NY, USA). For all tests, a p-value of < 0.05 was considered significant.

### Ethical statement

This study was conducted in accordance with the principles of the Declaration of Helsinki and was reviewed by our ethics committee. The study was not subject to the Medical Research Involving Human Subjects Act (WMO), and the requirement for informed consent was waived.

## Results

### Mechanical complications (short term)

Complications related to instability or soft tissues (Henderson type 1) occurred in nine (14%) reconstructions: three of the proximal humerus (3/10, 30%), four of the proximal femur (4/25, 16%), and two of the distal femur (2/14, 14%), after a median of 27 months (range 3–297). For reconstructions of the proximal femur, dislocation occurred in two total hip replacements (2/6, 33%) and in two bipolar hemiarthroplasties (2/19, 11%). Instability was treated with closed reduction in two APC (proximal femur), open reduction in four APC (two of the proximal femur, two of the proximal humerus), one rotator cuff repair (for proximal migration of the humeral head) and a tendon transfer (for instability of the knee). None failed as a result of type 1 complications.

Non-union (type 2) occurred in nine reconstructions (14%): four of the distal femur (4/14, 29%), two of the proximal humerus (2/10, 20%), one of the proximal tibia (1/15, 7%), and two of the proximal femur (2/25, 8%). Re-interventions for non-union were performed after a median of 11 months (range 6–30). Non-union was treated successfully with cancellous bone grafting and re-fixation in eight cases, one required revision of the reconstruction.

### Mechanical complications (mid- and long-term)

Aseptic loosening (type 2) occurred in seven reconstructions (11%): three of the proximal femur (3/25, 12%), one of the proximal humerus (1/10, 10%), one of the distal femur (1/14, 7%) and two of the proximal tibia (3/15, 13%), after a median of 14 years (range 2–18 years). Two cases of loosening of hip prostheses were successfully salvaged with cemented refixation, one cemented refixation in the tibia failed. Four were converted to an endoprosthesis and one was revised to a new APC. Loosening occurred in four cemented reconstructions and in three non-cemented reconstructions (HR_cs_ 0.54, 95% CI 0.12–2.43).

Structural complications (type 3) occurred in 16 reconstructions (25%). Allograft collapse was observed in 13 cases (20%), after a median of three years (range 1–15). One patient had an allograft fracture due to an adequate trauma, after three years. Eight collapses occurred in the proximal tibia (8/15, 53%), two in the proximal humerus (2/10, 20%), two in the distal femur (2/14, 14%), and one in the proximal femur (1/25, 4%). Two were treated conservatively because of acceptable function and absence of pain, the remaining eleven were either converted to an endoprosthesis (n = 10) or revised to a new APC (n = 2). Neither length of reconstruction, adjuvant therapies, osteosynthesis type, nor cementing technique were associated with the risk of collapse.

### Non-mechanical complications

Infection (type 4) occurred in five (8%) reconstructions, after a median of 2.6 months (0.4–2.9): two proximal humerus (2 of 10, 20%) and three of the proximal tibia (3 of 16, 19%). Two infections occurred after a secondary procedure. Infections that occurred within the first month (n = 2) were successfully eradicated with surgical debridement and systemic antibiotics. Three infections that occurred after more than one month postoperatively were revised to an endoprosthesis during a two-stage procedure. (Neo)adjuvant therapies were not associated with infection.

### Overall complications

Sixty-one percent of all patients experienced one or more complication(s), leading to an overall re-operation rate of 56%. Complications were most prevalent in the proximal tibia (14 of 15, 93%), followed by the proximal humerus (6 of 10, 60%), distal femur (8 of 14, 57%) and proximal femur (13 of 25, 52%).

### Cumulative incidence of reconstruction failure

**Fig. 3:**
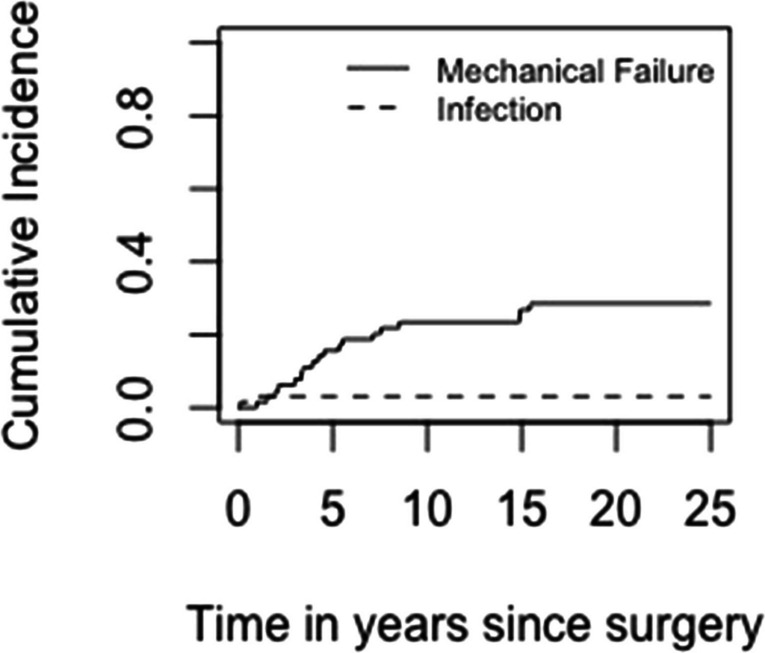
The cumulative incidence of implant failure for mechanical failure and infection

With failure for mechanical reasons (Henderson 1–3) as the end-point, the cumulative incidences of mechanical failure at five, ten and 25 years were 15.6% (95% CI 6.6–24.6), 21.9% (95% CI 11.6–32.1), and 28.6% (95% CI 17.2–39.9), respectively (Fig. 3). The cumulative incidences of failure due to infection at two months and 25 years were 1.6 (95% CI 0–4.6) and 3.1 (95% CI 0–7.4).

### Limb salvage

Limb salvage was achieved in 54 patients (95%). Ablative procedures were performed in three patients: two (4%) for infection, one because of tumour recurrence. At final follow-up, 40 patients (70%) had an APC reconstruction in situ and 14 reconstructions (25%) had been converted to an endoprosthesis.

## Discussion

Theoretically, allograft prosthetic composites offer a reconstructive option that combines the benefits of biological and endoprosthetic techniques. Several authors hypothesized that if an allograft survives the first years, it would provide for a durable reconstructive technique [22]. We conducted this long-term follow-up study to assess if APC indeed offer a reliable reconstructive technique on the long term.

We acknowledge limitations in our study. First, this is a retrospective cohort study with its inherent shortcomings. Second, one could prefer to report on a single localization, but numbers available are generally small. Therefore, with the limited numbers we had, we chose to report on APC as one group in order to create a general understanding of failure mechanisms of combining allografts and prostheses on the long term. Third, because of the long retrospective period, we were unable to collect data on functional outcomes.

Dislocations (Type 1) occurred in 16% of the proximal femoral reconstructions, which compares unfavourably with two previous studies, showing an aggregated dislocation risk of 3% in 76 reconstructions [17, 23]. In our study, two dislocations occurred after a secondary procedure that took place more than ten years after the primary procedure. Therefore, the high incidence of dislocation in our series might be a matter of follow up. The risk of dislocation appeared to be lower for hemiarthroplasties than for reconstructions with an acetabular cup. Previous authors also reported favourable results for hemiarthroplasties [24], although the indications for using an acetabular cup differed and results are therefore hard to compare. For reconstructions of the proximal humerus, we found a 30% risk of instability (two dislocations, one proximal migration). Despite the fact that we performed capsular imbrication and tendon reattachments, our results compare unfavourable with others, who reported instability in 0–9% of patients and dislocations in 4% [17, 25, 26]. Our results may indicate that the theoretical advantage of soft tissue reinsertion in APC may not be as pronounced as was previously believed.

The non-union (type 2) risk in our series (14%) is consistent with most series in literature (4–73%) [16, 17, 25]. Biau et al. reported a higher incidence of non-union (19/26, 73%) and attributed this to irradiation of allografts, lack of bone grafting and the use of chemotherapy [11]. Moon et al. reported nine non-unions in a study on 17 distal femoral APC (53%,) four of which could be salvaged with refixation and bone grafting [27]. In our series, eight of ten refixations resulted in union of the junction. Aggregated data from literature shows comparable results, with refixation and bone grafting being successful in 27 of 35 cases (77%) (Table 4).

**Table 4:** Overview of literature of allograft prosthetic composite reconstruction (at least 20 reconstructions)

Study Year of publication	Min et al. [17]	Biau et al. [28]	Abdeen et al. [25]	Donati et al. [29]	Biau et al. [11]	Farid et al. [16]	Donati et al. [23]	Hejna et al. [30]	Current study
	2015	2010	2009	2008	2007	2006	2002	1997	2026
No. of patients	28	32	36	62	26	20	27	24	57
No. of reconstructions	28	32	36	62	26	20	27	22	64
Localization									
Proximal femur	28 (100)	32 (100)				20 (100)	27 (100)	11 (50)	25 (39)
Distal femur								9 (41)	14 (21)
Proximal tibia				62 (100)	26 (100)			1 (5)	15 (23)
Proximal humerus			36 (100)					1 (5)	10 (15)
Follow-up (months)									
Mean	n/r	n/r	60	72	n/r	n/r	58	45	n/r
Median	56	68	n/r	n/r	128	78	n/r	n/r	294
Range	25–138	2–232	4–132	13–149	6–195	24–335	36–136	11–124	5–385
Surgery details									
Plates	n/r	n/r	n/r	6 (4)	n/r	10 (50)	n/r	22 (100)	54 (84)
Implant through osteotomy	n/r	32 (100)	36 (100)	62 (100)	26 (100)	20 (100)	27 (100)	22 (100)	25 (39)
Cement in host bone	28 (100)	32 (100)	28 (78)	1 (2)	26 (100)	18 (90)	4 (15)	n/r	29 (45)
Length									
Mean resection length (cm)	14	n/r	n/r	13	n/r	n/r	14	15	n/r
Median resection length (cm)	n/r	18	n/r	n/r	14	20	n/r	n/r	16
Range resection length	7–19	15–21	n/r	9–28	9–20	13–25	6–22	10–20	8–43
Outcomes									
Henderson 1 (instability)	0	n/r	1 (3)	n/r	n/r	2 (10)	0	n/r	9 (14)
Henderson 2 (non-union)	3 (12)	n/r	4 (11)	8 (13)	19 (73)	2 (10)	1 (4)	5 (23)	9 (14)
Henderson 2 (Loosening)	0	2 (6)	3 (8)	2 (3)	7 (27)	0	0	n/r	7 (11)
Henderson 3 (Hardware)	0	1 (3)	0	n/r	1 (4)	n/r	0	n/r	3 (5)
Henderson 3 (Collapse)	1 (4)	n/r	0	3 (5)	7 (27)	1 (5)	1 (4)	n/r	13 (20)
Henderson 4 (Infection)	0	4 (13)	0	15 (24)	6 (23)	1 (5)	1 (4)	n/r	5 (8)
Henderson 5 (tumour progression)	0	1 (3)	1 (3)	3 (5)	2 (8)	n/r	1 (4)	0	9 (14)
Conversion to tumourprosthesis	n/r	1 (3)	1 (3)	1 (2)	n/r	1 (5)	n/r	1 (5)	20 (31)
Re-operations	4 (1)	n/r	19 (7)	17 (27)	54 (14)	n/r	15(4)	n/r	56
Overall implant survival (15 years)	n/r	n/r	n/r	n/r	n/r	86	n/r	n/r	75
Overall implant survival (10 years)	n/r	n/r	88	68	33	86	n/r	n/r	78
Overall implant survival (5 years)	n/r	n/r	n/r	73	68	100	n/r	n/r	84

Aseptic loosening (type 2) occurred in seven (11%) reconstructions. Previous authors reported aseptic loosening in 0–27% of patients in cemented reconstructions [11, 15, 16, 25, 28]. We could not detect a significantly higher risk of loosening for uncemented implants. However, long-term stable fixation of uncemented implants relies on bone ongrowth, which presumably does not occur with avital allogeneic bone. Therefore, we believe that bone cement should be used. This series showed that cemented refixation can be used to successfully treat implant loosening in APC.

Allograft collapse (type 3) was observed in 20% of our reconstructions, predominantly of the proximal tibia. Previously reported incidences of fracture and collapse were 0–4% after two to five years follow up [17, 28], 0–5% after five to ten years follow up [16, 25, 29] and 27% after more than ten years follow up[11], suggesting a continuous risk over time. Our study confirmed this continuing risk. Combined with the ongoing risk of implant loosening, it appears that APCs do not offer reliable long-term mechanical stability. The limited potential for creeping substitution could be one of the explanations [8]. Ingrowth cannot take place at the articular side, as opposed to intercalary or hemicortical reconstructions.

Infections (type 4) occurred in eight percent of our reconstructions. Previously reported infection rates range between 4 and 12% for proximal humeral, proximal femoral and distal femoral reconstruction [17, 23, 25, 26, 28, 30]. On the other hand, in proximal tibial reconstructions, infection ranges between 23 and 24% [11, 29]. This might be explained by the limited possibilities for soft tissue coverage. Therefore, a gastrocnemius muscle flap rotation could be considered, as it has shown to decrease the risk of infection for endoprostheses [31]. From our study, it appeared that acute infections of APC can be treated successfully with surgical debridement and implant retention, combined with systemic antibiotics. Previously, only Donati reported on three successful debridement treatments (3/15, 20%) after infected APC of the proximal tibia [29].

The overall cumulative incidence of implant failure at ten years was 22%. Most previous studies used Kaplan–Meier survival analyses instead of competing risk analyses with corresponding cumulative incidences and previously used definitions of failure are inconsistent. Therefore, adequately comparing failure rates is challenging. However, at ten years follow up, reported reconstruction survival was 81–86% for the proximal femur [9, 26], 88% for the proximal humerus [25] and 33–68% for the proximal tibia [11, 29]. With a cumulative incidence of implant failure of 29% at 25 years, our results showed that implant failure continues to occur on the long term. In our experience, the use of APCs in reconstruction after tumour resection has decreased and its role has shifted to more specific cases. For example, we see that APCs still are particularly important in the paediatric population, where bone stock is prioritised for future revisions. The choice between APCs and modular endoprostheses should be individualised based on patient age, bone size and prognosis.

In conclusion, APC are associated with a considerable risk of complications, both on the short- and long-term. The lowest rate of complications occurred in the proximal femur, the highest in the proximal tibia. Non-union of allograft-host junctions is the main concern in the first years. Fractures and loosening are the predominant modes of failure on the long term, occurring as late as 15 years after the primary procedure. Because of the high risk of mechanical failure, that appears to persist on the long-term we are of the opinion that APC should not be used routinely for reconstruction after tumour resection.

## Data Availability

No datasets were generated or analysed during the current study.

## References

[1] Bielack SS, Kempf-Bielack B, Delling G, Exner GU, Flege S, Helmke K et al (2002) Prognostic factors in high-grade osteosarcoma of the extremities or trunk: an analysis of 1,702 patients treated on neoadjuvant cooperative osteosarcoma study group protocols. J Clin Oncol : Official J American Soc Clin Oncol 20(3):776–790. 10.1200/JCO.2002.20.3.776 10.1200/JCO.2002.20.3.776 11821461

[2] Arndt CA, Rose PS, Folpe AL, Laack NN (2012) Common musculoskeletal tumors of childhood and adolescence. Mayo Clin Proc 87(5):475–487. 10.1016/j.mayocp.2012.01.015 22560526 10.1016/j.mayocp.2012.01.015 PMC3538469

[3] Quan GM, Slavin JL, Schlicht SM, Smith PJ, Powell GJ, Choong PF (2005) Osteosarcoma near joints: assessment and implications. J Surg Oncol 91(3):159–166. 10.1002/jso.20268 16118770 10.1002/jso.20268

[4] Delloye C, Cornu O, Druez V, Barbier O (2007) Bone allografts: what they can offer and what they cannot. J Bone Joint Surg Br 89(5):574–579. 10.1302/0301-620X.89B5.19039 17540738 10.1302/0301-620X.89B5.19039

[5] Muscolo DL, Ayerza MA, Aponte-Tinao LA, Abalo E, Farfalli G (2007) Unicondylar osteoarticular allografts of the knee. J Bone Joint Surg Am 89(10):2137–2142. 10.2106/JBJS.F.01277 17908888 10.2106/JBJS.F.01277

[6] Ogilvie CM, Crawford EA, Hosalkar HS, King JJ, Lackman RD (2009) Long-term results for limb salvage with osteoarticular allograft reconstruction. Clin Orthop Relat Res 467(10):2685–2690. 10.1007/s11999-009-0726-9 19214644 10.1007/s11999-009-0726-9 PMC2745444

[7] Enneking WF, Campanacci DA (2001) Retrieved human allografts : a clinicopathological study. J Bone Joint Surg Am 83-A(7):971–986 11451965

[8] Bus MP, van de Sande MA, Taminiau AH, Dijkstra PD (2017) Is there still a role for osteoarticular allograft reconstruction in musculoskeletal tumour surgery? A long-term follow-up study of 38 patients and systematic review of the literature. Bone Joint J 99-B(4):522–530. 10.1302/0301-620X.99B4.BJJ-2016-0443.R2 28385943 10.1302/0301-620X.99B4.BJJ-2016-0443.R2

[9] Langlais F, Lambotte JC, Collin P, Thomazeau H (2003) Long-term results of allograft composite total hip prostheses for tumors. Clin Orthop Relat Res 414:197–211. 10.1097/01.blo.0000079270.91782.23 10.1097/01.blo.0000079270.91782.23 12966294

[10] Campanacci L, Ali N, Casanova JM, Kreshak J, Manfrini M (2015) Resurfaced allograft-prosthetic composite for proximal tibial reconstruction in children: intermediate-term results of an original technique. J Bone Joint Surg Am 97(3):241–250. 10.2106/JBJS.N.00447 25653325 10.2106/JBJS.N.00447

[11] Biau DJ, Dumaine V, Babinet A, Tomeno B, Anract P (2007) Allograft-prosthesis composites after bone tumor resection at the proximal tibia. Clin Orthop Relat Res 456:211–217. 10.1097/BLO.0b013e31802ba478 17091014 10.1097/BLO.0b013e31802ba478

[12] Jeys LM, Kulkarni A, Grimer RJ, Carter SR, Tillman RM, Abudu A (2008) Endoprosthetic reconstruction for the treatment of musculoskeletal tumors of the appendicular skeleton and pelvis. J Bone Joint Surg Am 90(6):1265–1271. 10.2106/JBJS.F.01324 18519320 10.2106/JBJS.F.01324

[13] Bus MP, van de Sande MA, Fiocco M, Schaap GR, Bramer JA, Dijkstra PD (2015) What Are the Long-term Results of MUTARS Modular Endoprostheses for Reconstruction of Tumor Resection of the Distal Femur and Proximal Tibia? Clin Orthop Relat Res. 10.1007/s11999-015-4644-8 10.1007/s11999-015-4644-8 PMC5289150 26649558

[14] Grimer RJ, Aydin BK, Wafa H, Carter SR, Jeys L, Abudu A et al (2016) Very long-term outcomes after endoprosthetic replacement for malignant tumours of bone. Bone Joint J 98-b(6):857–864. 10.1302/0301-620x.98b6.37417 27235533 10.1302/0301-620X.98B6.37417

[15] Donati D, Colangeli M, Colangeli S, Di BC, Mercuri M (2008) Allograft-prosthetic composite in the proximal tibia after bone tumor resection. Clin Orthop Relat Res 466(2):459–465. 10.1007/s11999-007-0055-9[doi] 10.1007/s11999-007-0055-9 PMC2505118 18196432

[16] Farid Y, Lin PP, Lewis VO, Yasko AW (2006) Endoprosthetic and allograft-prosthetic composite reconstruction of the proximal femur for bone neoplasms. Clin Orthop Relat Res.;442:223–9 00003086–200601000–00036 [pii] 10.1097/01.blo.0000181491.39048.fe 16394765

[17] Min L, Tang F, Duan H, Zhou Y, Zhang WL, Shi R et al (2015) Cemented allograft-prosthesis composite reconstruction for the proximal femur tumor. Onco Targets Ther 8:2261–2269. 10.2147/OTT.S85788 26345329 10.2147/OTT.S85788 PMC4556043

[18] Henderson ER, O’Connor MI, Ruggieri P, Windhager R, Funovics PT, Gibbons CL et al (2014) Classification of failure of limb salvage after reconstructive surgery for bone tumours : a modified system including biological and expandable reconstructions. Bone Joint J 96-B(11):1436–40. 10.1302/0301-620X.96B11.34747 25371453 10.1302/0301-620X.96B11.34747

[19] Bus MP, Dijkstra PD, van de Sande MA, Taminiau AH, Schreuder HW, Jutte PC et al (2014) Intercalary allograft reconstructions following resection of primary bone tumors: a nationwide multicenter study. J Bone Joint Surg Am 96(4):e26. 10.2106/JBJS.M.00655 24553895 10.2106/JBJS.M.00655

[20] de Wree LC, Fiocco M, Putter H (2010) The mstate package for estimation and prediction in non- and semi-parametric multi-state and competing risks models. Comput Methods Programs Biomed 99(3):261–274. 10.1016/j.cmpb.2010.01.001 20227129 10.1016/j.cmpb.2010.01.001

[21] Core Team R (2007) R: A language and environment for statistical computing v. 2.7. 1. R Foundation for Statistical Computing, Vienna, Austria

[22] Ortiz-Cruz E, Gebhardt MC, Jennings LC, Springfield DS, Mankin HJ (1997) The results of transplantation of intercalary allografts after resection of tumors. A long-term follow-up study. J Bone Joint Surg Am 79(1):97–106 9010190 10.2106/00004623-199701000-00010

[23] Donati D, Giacomini S, Gozzi E, Mercuri M (2002) Proximal femur reconstruction by an allograft prosthesis composite. Clin Orthop Relat Res 394:192–200 10.1097/00003086-200201000-00023 11795733

[24] Chandrasekar CR, Grimer RJ, Carter SR, Tillman RM, Abudu A, Buckley L (2009) Modular endoprosthetic replacement for tumours of the proximal femur. J Bone Joint Surg Br 91(1):108–112. 10.1302/0301-620X.91B1.20448 19092014 10.1302/0301-620X.91B1.20448

[25] Abdeen A, Hoang BH, Athanasian EA, Morris CD, Boland PJ, Healey HJ (2009) Allograft-prosthesis composite reconstruction of the proximal part of the humerus: functional outcome and survivorship. J Bone Joint Surg Am 91(10):2406–2415. 10.2106/JBJS.H.00815 19797576 10.2106/JBJS.H.00815

[26] Farid Y, Lin PP, Lewis VO, Yasko AW (2006) Endoprosthetic and allograft-prosthetic composite reconstruction of the proximal femur for bone neoplasms. Clin Orthop Relat Res 442:223–229 16394765 10.1097/01.blo.0000181491.39048.fe

[27] Moon BS, Gilbert NF, Cannon CP, Lin PP, Lewis VO (2013) Distal femur allograft prosthetic composite reconstruction for short proximal femur segments following tumor resection. Adv Orthop 2013:397456. 10.1155/2013/397456 24349792 10.1155/2013/397456 PMC3855933

[28] Biau DJ, Larousserie F, Thevenin F, Piperno-Neumann S, Anract P (2010) Results of 32 allograft-prosthesis composite reconstructions of the proximal femur. Clin Orthop Relat Res 468(3):834–845. 10.1007/s11999-009-1132-z 19851817 10.1007/s11999-009-1132-z PMC2816772

[29] Donati D, Colangeli M, Colangeli S, Di Bella C, Mercuri M (2008) Allograft-prosthetic composite in the proximal tibia after bone tumor resection. Clin Orthop Relat Res 466(2):459–465. 10.1007/s11999-007-0055-9 18196432 10.1007/s11999-007-0055-9 PMC2505118

[30] Hejna MJ, Gitelis S (1997) Allograft prosthetic composite replacement for bone tumors. Semin Surg Oncol 13(1):18–24 9025178 10.1002/(sici)1098-2388(199701/02)13:1<18::aid-ssu4>3.0.co;2-7

[31] Myers GJ, Abudu AT, Carter SR, Tillman RM, Grimer RJ (2007) Endoprosthetic replacement of the distal femur for bone tumours: long-term results. J Bone Joint Surg Br 89(4):521–526. 10.1302/0301-620X.89B4.18631 17463123 10.1302/0301-620X.89B4.18631

[32] Wolters U, Wolf T, Stutzer H, Schroder T (1996) ASA classification and perioperative variables as predictors of postoperative outcome. Br J Anaesth 77(2):217–222 8881629 10.1093/bja/77.2.217

